# Ipsilateral Hemiparesis in a Patient With Existing Contralateral Hemiparesis: A Case Report of a Rare Presentation of Ischemic Stroke

**DOI:** 10.7759/cureus.37069

**Published:** 2023-04-03

**Authors:** Kavya Mala, Ganaraja V Harikrishna, Vivek Bhat, Suresha Kodapala

**Affiliations:** 1 Neurology, Vydehi Institute of Medical Sciences and Research Centre, Bangalore, IND; 2 Internal Medicine, St. John's Medical College, Bangalore, IND

**Keywords:** functional mri, rehabilitation, corticospinal tract, cortical reorganization, post-stroke recovery

## Abstract

Supratentorial strokes causing ipsilateral hemiparesis (ILH) are rare. We report a middle-aged male with multiple atherosclerotic risk factors, who had previously suffered a right-hemispheric stroke that caused left hemiplegia. Subsequently, he presented with worsening left-sided hemiplegia, with imaging revealing a left-hemispheric stroke. Diffusion tensor tract imaging showed crossed motor tracts, with disruption of the left-sided pyramidal tract. During his stay, he developed right hemiplegia due to the expansion of the same left-hemispheric infarct. Potential mechanisms for ILH in a stroke include injury to reorganized tracts following an initial insult and congenitally uncrossed motor tracts. In our patient, after his first stroke, the left hemisphere likely assumed greater ipsilateral motor control, causing ILH after the recent stroke. Our case adds to the literature on this interesting phenomenon and provides further insight into post-stroke recovery.

## Introduction

Supratentorial strokes classically result in contralateral neurologic deficits, due to the well-described ‘crossing’ of the corticospinal tracts (CSTs) [1]. Ipsilateral hemiparesis (ILH) due to cerebrovascular disease is incredibly rare. We report a left-hemispheric infarct that worsened existing left-sided hemiplegia from a prior right-sided stroke, with the later development of right-sided hemiplegia due to the expansion of the same left-hemispheric infarct.

## Case presentation

Our patient was a 56-year-old male, who was a known case of type-2 diabetes mellitus, and hypertension, with history of nicotine abuse for the last 40 years. He had left hemiplegia since the last three years, with residual weakness (modified Rankin score (mRS) of 2), and presented with complaints of sudden onset worsening of weakness of left upper and lower extremities (LUE, LLE), deviation of angle of mouth to the right, and difficulty in pronouncing words clearly, since approximately six hours. On examination, he had dysarthria, left upper-motor-neuron type facial weakness, and spasticity in the LUE and LLE. He had left-sided hemiplegia, with Medical Research Council (MRC) scores of 2/5 and 1/5 in the proximal and distal LUE and LLE, respectively, and 5/5 in the right upper and lower extremities (RUE, RLE). He had no sensory or cerebellar deficits.

**Figure 1:**
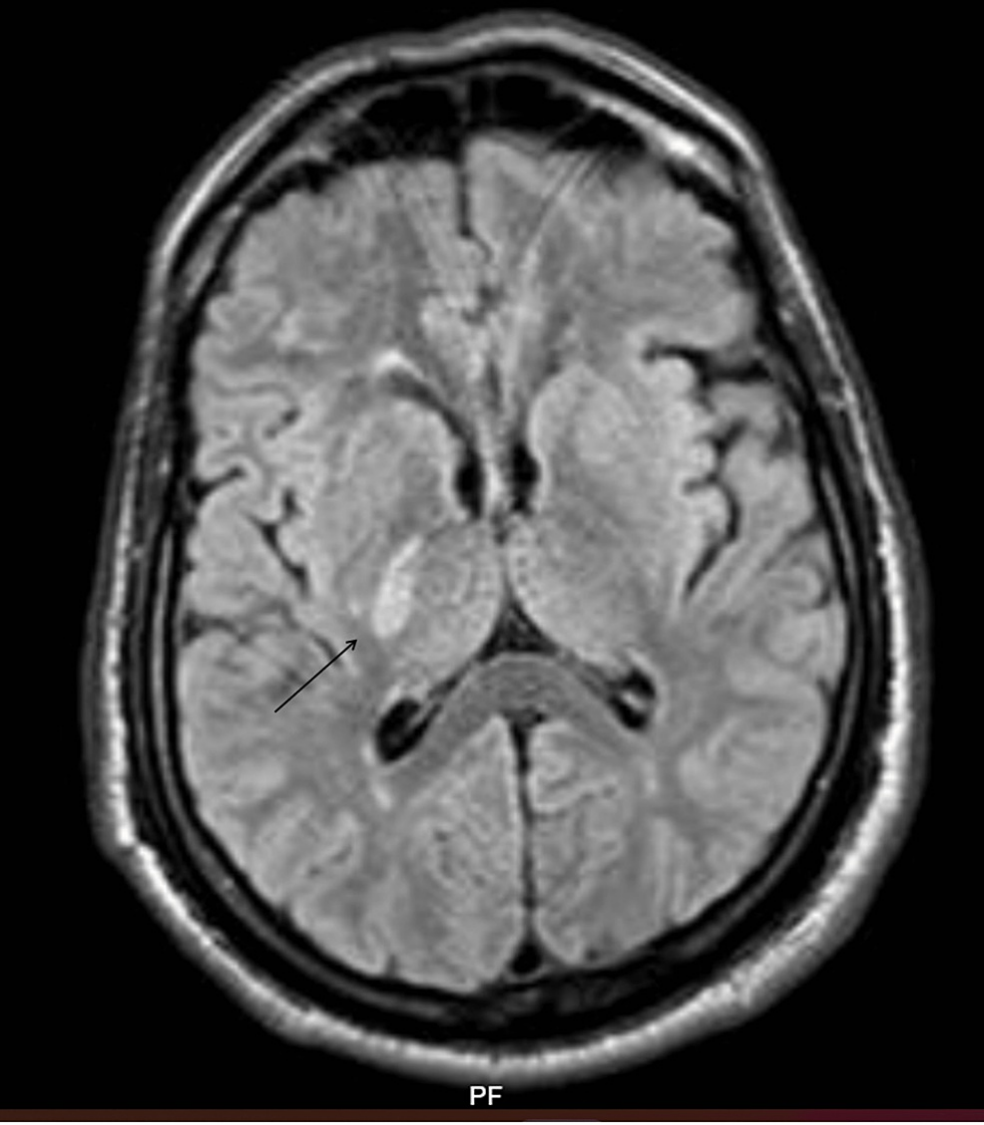
MRI brain during first stroke, three years back showing FLAIR hyperintensity in right capsuloganglionic region (black arrow). MRI: magnetic resonance imaging; FLAIR: fluid-attenuated inversion recovery.

Brain magnetic resonance imaging (MRI) from three years ago showed an acute infarct in the right capsuloganglionic region (Figure 1).

**Figure 2:**
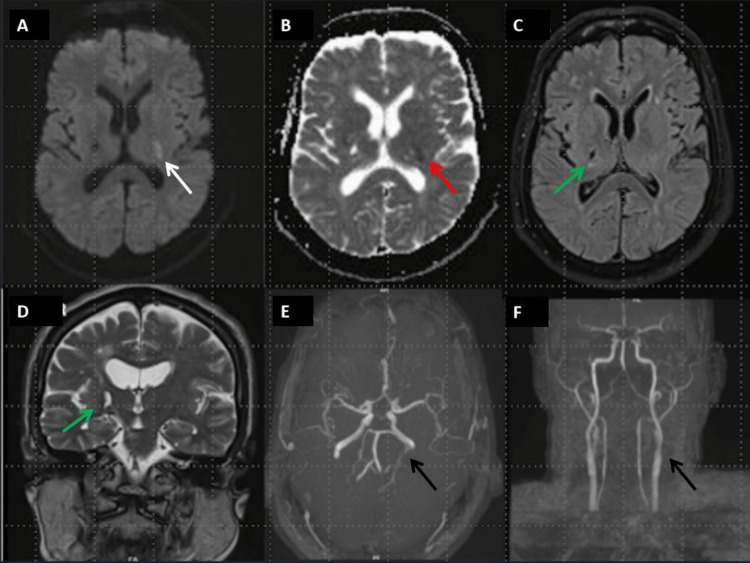
MRI brain showing (A, B) diffusion restriction in left capsuloganglionic region on DWI (white arrow), with corresponding ADC (red arrow) suggesting acute infarct; (C, D) FLAIR axial sequence showing hypointensity in right capsuloganglionic region and hyperintensity in T2 coronal sequence suggesting gliosis secondary to old stroke (green arrows); (E, F) MRA showing normal cranial vessels (black arrows). MRI: magnetic resonance imaging; DWI: diffusion-weighted imaging; ADC: apparent diffusion coefficient; FLAIR: fluid-attenuated inversion recovery; MRA: magnetic resonance angiography

Brain MRI at the time of presentation, with acute worsening of left-sided weakness, showed an acute infarct in the left posterior limb of the internal capsule and evidence of a chronic infarct in the right capsuloganglionic region (Figure 2).

**Figure 3:**
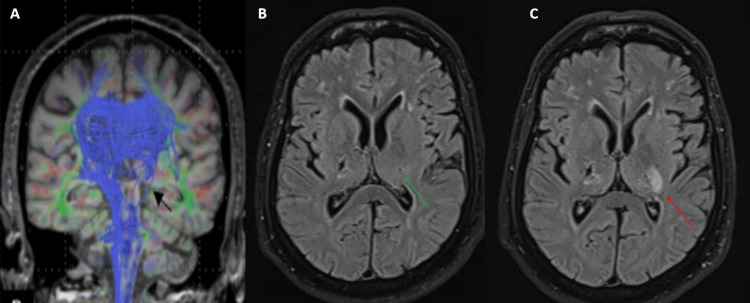
(A) Diffusion tensor imaging tractography showing disruption of left-sided corticospinal fibers (black arrow); (B) Brain MRI scan FLAIR sequence on first day of presentation showing hyperintensity in left capsule-ganglionic region (green arrow); (C) repeat imaging of the same region after four days showing lesion expansion (red arrow). MRI: magnetic resonance imaging; FLAIR: fluid-attenuated inversion recovery

Diffusion tensor tract imaging showed interruption and attenuation of the left corticospinal tract from left posterior limb of internal capsule and cerebral peduncles, up to medullary pyramids (Figure 3A). Neck vessel doppler ultrasound showed no significant luminal stenosis, while cardiac workup and MRI spine were normal. Subsequently, four days into his hospital stay, he developed weakness and ataxia of his RUE and RLE. Repeat neurological examination revealed MRC power of 4/5 in the distal RUE and RLE, with impaired finger-nose test and knee-heel test, suggesting ataxic hemiparesis. Repeat brain MRI revealed expansion of the left capsule-ganglionic infarct (Figure 3B, 3C).

Finally, he was diagnosed with acute lacunar infarct in the left posterior limb of internal capsule causing worsening of ipsilateral left hemiplegia, with expansion of the same infarct causing right ataxic hemiparesis. He was treated with appropriate antiplatelets and supportive therapy and was discharged after 10 days with residual quadriparesis (mRS-4).

## Discussion

ILH has been reported to occur in 0.17% of ischemic strokes [2]. Reported mechanisms include the presence of congenitally uncrossed CSTs, transhemispheric diaschisis from the affected side, and cerebrovascular injury after post-stroke neuronal reorganization [2,3]. Interestingly, these mechanisms may coexist; for example, Tan et al. reported bilateral cortical motor activation after a stroke in a patient with congenitally uncrossed CSTs [4].

In our patient, we believe that injury to reorganized neurons, likely in the posterior limb of the internal capsule, carrying the CST was responsible for his ILH. This is because his initial stroke, three years prior to consulting us, caused contralateral weakness. Further, diffusion tensor imaging did not reveal the presence of uncrossed CSTs. In the available literature, this appears to be the most common mechanism of ILH in strokes [1,2]. Most such reported cases occurred in patients with prior strokes that caused weakness contralateral to the lesion. Saada et al. reported two cases of ILH after a stroke, with clinical signs pointing to uncrossed CSTs [1]. Similarly, Song et al. reported two cases and Ago et al. reported one case of ILH after older strokes that caused contralateral weakness; functional MRI (fMRI) revealed ipsilateral activation with motion of the paretic side, suggesting cortical reorganization [5,6]. Inatomi et al. analyzed over 8000 cases of ischemic stroke in their center and found that out of 14 patients with ILH, most had crossed CSTs [2].

Among patients with ILH and prior history of stroke, Inatomi et al. identified three major common characteristics: (1) the prior stroke was contralateral to the recent stroke, and caused paresis contralateral to the lesion; (2) the recent strokes causing ILH were located along the CST; and (3) patients had a crossed CST on functional imaging [2]. Contrastingly, patients with congenitally uncrossed CSTs typically have associated structural disease such as congenital scoliosis, arachnoid cysts, or agenesis of the corpus callosum [1,2,4,7]. Our case met all three characteristics reported by Inatomi et al. and lacked any other structural neurologic disease.

Post-stroke recovery has been elucidated using positron emission tomography (PET) or fMRI, and occurs through three major processes: synaptogenesis in the peri-infarctional areas, recruitment of secondary motor areas, and finally, cortical reorganization in the unaffected hemisphere [6,8,9]. In our patient, we hypothesize that after his first stroke in the right hemisphere, his left hemisphere underwent reorganization to regain some left-sided motor control. These reorganized neurons were impaired in his recent stroke, with the subsequent infarct expansion damaging the original, non-reorganized neurons, that controlled right-sided motor function. These original neurons likely belonged to the CST and intermingled corticocerebellar fibers, leading to his ataxic hemiparesis [10]. Ideally, PET or fMRI could have delineated the exact recovery and pathophysiology of our patient’s clinical course [11-13]; however, due to resource limitations, this was not feasible.

## Conclusions

We describe a case of ILH after a stroke, due to damage to corticospinal tracts that reorganized after a prior stroke. Our report adds valuable literature regarding cortical reorganization after a stroke, and post-stroke recovery. Further large studies and reports are necessary to elucidate this phenomenon.
